# Clonal Hematopoiesis as a Predictor of Immune Toxicity and Efficacy After Chimeric Antigen Receptor T-cell Therapy: a Systematic Review and Meta-Analysis

**DOI:** 10.51894/001c.164015

**Published:** 2026-07-31

**Authors:** Claire Bischel, Adam Bowen, Kristina Golovataya, Claire Russell, Muhammad Saleem, Sarah Gergis, Katlyn Wendel, Lakshmi Thulluri, Stephen Caucci, Sanjay Rao Gergal Gopalkrishna Rao, Kirolos Gergis, Borys Hrinczenko

**Affiliations:** 1 McLaren Greater Lansing Hospital

## 13

### Background

Clonal hematopoiesis of indeterminate potential (CHIP), a form of clonal hematopoiesis (CH), is linked to pro-inflammatory myeloid phenotypes and may influence immune toxicity and efficacy after chimeric antigen receptor (CAR) T-cell therapy. We performed a systematic review and meta-analysis to quantify associations between sequencing-defined CH and clinically significant cytokine release syndrome (CRS), immune effector cell-associated neurotoxicity syndrome (ICANS), and treatment response after CAR T-cell therapy.

### Methods

We searched PubMed, Embase, CENTRAL, and Web of Science from inception to February 2026 for studies reporting sequencing-defined CH in CAR T recipients and pooled outcomes by CH status using random-effects Mantel-Haenszel models. Primary endpoints were severe CRS and severe ICANS (ASTCT grade ≥3).

### Results

Nine observational cohorts (n=869) were included. CH status was available for 736 patients (84.7%), with prevalence 24%–56% across study-specific variant allele fraction (VAF) thresholds (most commonly ≥2%). Risk of bias was moderate in seven studies and serious in two. Across four cohorts (n=360), CH was not associated with severe CRS (RR 0.72, 95% CI 0.24–2.13) or severe ICANS (RR 1.16, 95% CI 0.48–2.81). Secondary toxicity outcomes were also not significantly different for CRS grade ≥2 (RR 1.04, 95% CI 0.73–1.48) or any-grade ICANS (RR 0.89, 95% CI 0.45–1.74). In prespecified analyses restricted to CD19-directed products, CH was associated with higher severe ICANS risk (RR 1.66, 95% CI 1.03-2.65), although estimates were sensitive to study-specific CH/CHIP definitions. CH-positive status was associated with modestly higher overall response (RR 1.10, 95% CI 1.01–1.21) and complete response (RR 1.24, 95% CI 1.01–1.52).

### Conclusions

Across this systematic review and meta-analysis of nine observational cohorts, CH was not associated with increased risk of clinically significant CRS or ICANS after CAR T-cell therapy. Prospective, standardized studies incorporating clone size and genotype, uniform CRS/ICANS assessment, and integrated biomarker and CAR expansion profiling are needed before CH can be meaningfully incorporated into clinical risk stratification or treatment decision-making.

